# The Relationship between Young Children’s Graphomotor Skills and Their Environment: A Cross-Sectional Study

**DOI:** 10.3390/ijerph20021338

**Published:** 2023-01-11

**Authors:** Rachel-Tzofia Sinvani, Anat Golos, Stav Ben Zagmi, Yafit Gilboa

**Affiliations:** 1 School of Occupational Therapy, Faculty of Medicine, Hebrew University of Jerusalem, Jerusalem 9124001, Israel; 2 Child Development Center, “Meuhedet” Health Maintenance Organization, Ashdod 7727408, Israel

**Keywords:** bioecological model, school readiness, educational approach, socioeconomic status, motor skills, typically developing

## Abstract

The current study aimed to examine the unique contribution of personal and environmental factors to explain graphomotor skills in typically developing preschoolers and first-year elementary school students. A convenience sample of 136 Israeli children aged three–seven years was recruited. Graphomotor skills were assessed using the Gilboa Functional Test (GIFT); personal and environmental factors were assessed using a demographic questionnaire and the Home Literacy Experiences Questionnaire (HLEQ). A hierarchical multiple linear regression analysis revealed that home literacy and educational approach accounted for 43.1% of the variance of graphomotor skills (R^2^ = 40.4, *p* < 0.000), each providing a unique contribution to the explained variance after controlling for age, gender, and spoken language. Generally, our results supported the bioecological model, with proximal factors (home literacy and educational approach) having a greater influence on child graphomotor skills than distal factors (parental socioeconomic and immigration status). By highlighting the role of environmental factors in graphomotor development, these results can be used as a conceptual framework for developing early intervention programs.

## 1. Introduction

The term “graphomotor skills” refers to a subset of fine-motor skills that directly involve the operation of an ordinary pencil [1]. Graphomotor tasks are the combined outcome of various cognitive and musculoskeletal processes, including visual-spatial perception, size discrimination, visual retrieval, and orientation discrimination [2]. Moreover, motor skills, including graphomotor abilities, may be prerequisites for daily life functioning and for other developmental skills, including perception, cognition [3], and language [4]. Graphomotor skills that involve writing implements are important predictors of later academic achievement and for everyday participation [1,2,5,6]. However, although considered eminent in motor development, graphomotor belongs to the under-researched specific developmental skills of preschool children [7].

Preschool years represent a time of great curiosity and tendency to play, with stimulation offering dynamic opportunities to practice and explore, they likewise constitute a period of great physical activity during development [8,9]. During these years, a child’s repertoire of motor functions expands substantially [10]. In addition to age, gender is also associated with children’s developing motor skills, though relevant empirical data are inconsistent and lacking. The contradictory findings suggest that gender differences in motor performance are either too small to be detected, that they are restricted to specific motor skills, some of which favor girls and some others boys [11], or influenced by gender cultural expectations [12]. It has been shown that girls perform fine motor tasks better than boys in early childhood [10,12,13], as well as learn novel tasks earlier [14,15], while boys outperform girls in tasks requiring object control (like tossing a ball) [16,17]. Environmental factors such as parental expectations about appropriate and suitable motor tasks may contribute to these gender differences [18]. As with other developmental domains, motor development cannot be studied in isolation, divorced from the environmental and sociocultural conditions in which it occurs [19]. Moreover, motor development is shaped through the interaction of a child’s maturation and environmental experiences and thus a better understanding of this developmental process should take into account both factors [11]. While most research has focused on biological and psychological aspects of motor development, only a few studies have examined environmental factors [20,21,22]. In its earliest inception, Urie Bronfenbrenner’s ecological model [23] gave importance to place and formulated an individual’s environment as a nested, interrelated system. Further, Bronfenbrenner refined and revised his theory to what would later become known as the bioecological model. During this time, more concern was given to differentiating between the concepts of environment, personal characteristics, proximal process, and the concept of time as they relate to human development [24,25].

The bioecological model is conceived as a set of layers, each inside the next. The first inner system, called the microsystem, refers to the immediate environment in which the child lives. Microsystems include any immediate relationships or organizations the child interacts with, such as children’s direct interaction with parents. Indeed, many studies have considered the family system to be a key influence on the child’s development because of its close relationship with the child [26,27,28]. It is generally agreed that in this early period, children’s ongoing contact with an environment that encourages physical activity may facilitate normal development and offer opportunities for more exploration and interaction [8,9,29]. A previous study has demonstrated that the immediate environment where a child is raised influences the development of motor skills in the early years of life [29]. For example, a study that examined differences in motor developmental profiles among 50 preschool-aged children found that family-reared children had better motor performance, compared to children living in institutions [29]. 

As part of the microsystem, we included in our current investigation components that illustrate the physical richness of the home environment (housing density) as well as the home literacy environment. Home literacy environment refers to activities undertaken by family members at home that relate to literacy learning [30], as well as the literacy resources in the home and parental attitudes toward literacy [31]. Our motivation to include the literacy component in the immediate system stemmed from the established association between both those developmental skills, when graphomotor skills play a key role in the development of early literacy [28,32].

In the current study, we were specifically interested in the extent to which parents involved their children in various informal print-related activities, and to what extent these were child-initiated [33]. Informal literacy interactions include a variety of activities in which parents read to their child or direct their attention to print in the environment, such as advertisements or street names; such daily shared reading experiences between parents and children [34].

The second system, following the bioecological model known as exosystem, has an indirect effect on an individual’s developmental outcome and is the setting in which the individual does not actively participate. With regard to the exosystem, we chose to include the educational approach to which preschoolers were exposed. Chow and Louie (2013) examined the influence of the preschool types of motor skill performance in 239 children aged 3 to 6.5 years. Their results indicated that children from private preschools performed better in locomotor skills than those from public preschools. However, no difference was found in object control skills [35]. Here, we analyzed the influences of the preschool curricula, comparing the nation’s standard curricula (which has been taught in the public schools) with those of private schools based on Waldorf’s philosophy. The Waldorf schools, alternately known as “Anthroposophic Education” [36], are based on a holistic child-centered understanding of the human being and defer to “the will” of the kindergarten child who learns through imitation and play [37]. The Waldorf schools are designed to meet the needs and competencies of individuals, while public school curricula follow generally uniform, developmentally appropriate requirements [36]. Noticeably, support for Waldorf grew very rapidly, and by the 2020s it had become the most popular secular school movement in Israel [38]. 

Bronfenbrenner’s final level is the macrosystem. This most distal system involves society and includes cultural values and describes the economic conditions under which families are living [23,26]. Furthermore, the interrelations among these nested environments allow for the examination of how patterns of interactions within these systems influence each other and affect individuals’ developmental outcomes [23], such as parents’ socioeconomic status (SES) and cultural influences on the child’s other social systems [39,40]. Indeed, differences among cultural groups are often noted in the sequence and pace of children’s development in various areas, such as motor skills. Further, a part of the variability in children’s development may also be explained by social-cultural values and ideologies that often influence educational practices and policies in general [12]. Taking together the potential influence of both socio-cultural backgrounds, we were therefore motivated to study two more distal components constituting the macrosystem: one reflecting parental cultural background (as represented by immigration history), and one related to socioeconomic status (SES). Here, maternal education was used as a proxy for SES because it has been positively correlated with early childhood motor-skill measures. Moreover, maternal education has been one of the most consistent and reliable demographic details obtainable [41].

To summarize, given that motor competence constitutes a significant developmental challenge during preschool years [11], the present study was designed to investigate the effect of personal and environmental factors on preschool children’s specific graphomotor skills. While there have been extensive studies of how environments influence social, cognitive, and behavioral development [42,43,44,45], little is still known about how those factors influence graphomotor abilities. In order to fill this gap, we extend the available literature to the study of children’s graphomotor skills, by applying the bioecological theoretical framework and using ecologically valid assessment tools. We hypothesized that the associations of graphomotor skills with environmental factors would be influenced by the child’s personal characteristics (age, gender, and spoken language) and that they would be differentiated by the relevant environmental systems, from proximal to distal (i.e., physical home and literacy environment, preschool curricula, the immigration status of parents, and SES). Specifically, the dependent variable in our current study was therefore graphomotor performance, while independent variables were gender, age, spoken language, home literacy experience, educational approach, immigration status, and SES. 

## 2. Materials and Methods

### 2.1. Participants and Procedure

This study employed a cross-sectional design and a convenience sampling of typically developing preschoolers and first-year elementary school students from all over Israel. Recruitment was performed through social networking sites (WhatsApp and Facebook Groups). Children with severe cognitive challenges such as intellectual disabilities, psychiatric disorders, total hearing or visual loss, or severe manual dexterity issues (unable to carry out the test) were excluded.

Data were collected from 136 children (66 boys and 70 girls) between the ages of 3–7 years (M = 5.15 years, SD = 1.06). Table 1 contains all the demographic characteristics of the sample. The majority of the children (*n* = 120, 88.2%) were right-handed and attended public schools (*n* = 92, 71%). The SES was determined by years of maternal education (12 years and below, or above 12 years). Most mothers (80.7%) had received an education of over 12 years, and most of the children (72%) were born in Israel, but their first language was not Hebrew (e.g., English, Arabic, Russian).

One of each child’s parents provided written informed consent, and all of the children provided oral consent. A snowball sampling method, using word of mouth and social media platforms, was used to collect data from February through June 2018. While one of the parents filled out the questionnaires, the child performed the GIFT test. The raters in our study were all third-year occupational therapy students who had been trained in the GIFT’s administration and scoring. Testing took about 15 to 20 min and was carried out at the child’s home.

### 2.2. Measures 

#### 2.2.1. Demographic Questionnaire

Parents were asked to complete a demographic questionnaire designed for this study, to gather the following information: child’s gender, age, hand dominance, education, and language(s) spoken at home. With regard to the child’s education, parents were asked to indicate their child’s educational settings (daycare, preschool, and school) and educational approach (national vs. Waldorf). Furthermore, parents were asked about their educational history and housing density (number of people per room), with lower housing density corresponding to a richer physical environment [46,47].

#### 2.2.2. The Gilboa Functional Test (GIFT; [48])

The GIFT is a norm-referenced instrument for use with children 3–7 years of age, which measures performance skills based on everyday functioning. The GIFT includes 5–8 items (depending on the child’s age) using a range of graphomotor skills that every child may demonstrate in preschool and first grade. The items include copying geometric figures, coloring within lines, cutting with scissors, drawing a person, writing the child’s first name, writing the alphabet, writing the numbers 1–10, and phonetic writing. The GIFT consists of four age-designated versions based on the normal development of these eight activities: 3–4, 4–5, 5–6, and 6–7. The total score is calculated by summing all test items, with a higher score indicating better performance on the test. In order to allow comparisons between different versions, age-adapted total scores are converted into a standard score format (z score: mean = 0, SD = 1). Psychometric properties of the GIFT were assessed in a community-based sample of 611 preschoolers and first-year elementary school students, from diverse cultural and educational backgrounds. Based on the Pearson correlation, the GIFT demonstrated excellent test-retest reliability (r = 0.95, *p* < 0.01) and inter-rater reliability (r = 0.94, *p* < 0.01). Significant correlations were found with the Beery–Buktenica Developmental Test [49] for visual-motor integration and motor coordination (r = 0.32, *p* < 0.05; and r = 0.33, *p* < 0.05, respectively); with the Movement Assessment Battery for Children (M-ABC; [50]), r = −0.364, *p* < 0.05; and with the Developmental Coordination Disorder Questionnaire (DCDQ; [51]), r = 0.41, *p* < 0.01; all of which indicate good concurrent validity [48]. 

#### 2.2.3. Home Literacy Experiences Questionnaire (HLEQ; [33])

The purpose of this questionnaire is to assess the extent to which parents involved their children in various informal print-related activities and to provide a measure of the child’s independent pursuit of literacy activities. It contains 21 items that mention different literacy activities (for example, listening to books that parents read, and visiting local libraries). Parents are asked to rate how often their children were involved in those activities (i.e., frequency), on a scale from 1 (“never”) to 6 (“very often”), and the likelihood that the activity was initiated by the parents or by the child (i.e., independency), on a scale from 1 (“always initiated by the parent”) to 5 (“mostly initiated by the child”). In each scale, an average is computed, with higher scores indicating more frequent participation and greater independence of the child. 

With the authors’ approval, the questionnaire was translated into Hebrew for the current study. The Hebrew version was found to have a high-reliability coefficient (frequency scale: Cronbach’s alpha = 0.91, independence scale: alpha = 0.92). Previous studies have provided psychometric support for the reliability and validity of the HLEQ [52]. Levy et al. (2006) conducted a factor analysis of the HLEQ using 346 children aged 4–6 years. Analysis of the frequency scale resulted in seven principal components accounting for 58% of the variance. A principal component analysis of the independence scale resulted in four factors cumulatively accounting for 61% of the variance [33].

### 2.3. Data Analysis

The SPSS Version 25 was used for all statistical analyses. GIFT and HLEQ scores, as well as the demographic variables, were tabulated and analyzed. We used z-standard scoring to evaluate the GIFT scores across all four protocols. The GIFT scores of different groups (gender, educational approach, immigration status) were compared using the independent sample *t*-test, or Welch’s *t*-test for unequal variances when sample sizes differed. We calculated Pearson correlations between GIFT scores and HLEQ scores, as well as years of maternal education and housing density. We also conducted a one-way Welch’s ANOVA with Bonferroni post-hoc testing to compare the GIFT scores between groups (by spoken language). Hierarchical multiple regression analysis was conducted to determine GIFT total score predictors. After controlling for personal factors in block one (age, gender, and language spoken at home), the remaining blocks included the different environmental factors from proximal to distal. Block two included the microsystem, block three included the exosystem, and block four included the macrosystem variables. Alpha level was set at 0.05. 

## 3. Results

Table 2 provides descriptive statistics for the GIFT results for each age group, as well as scores obtained in the HLEQ. Based on the bioecological model, the results are presented from proximal to distal systems. 

### 3.1. Personal Factors

#### 3.1.1. Age

In the age groups of 3–4 and 4–5, Pearson correlation analysis demonstrated a significant positive relationship between age and GIFT total scores. However, the older age groups did not show a significant correlation (see Table 2).

#### 3.1.2. Gender

Using an independent *t*-test, no differences were found (t_(1,134)_ = −1.74, *p* > 0.05) between boys’ and girls’ GIFT total scores (see Table 3).

#### 3.1.3. Spoken Language

Welch’s ANOVA test showed significant differences (F_(5,134)_ = 4.52, *p* < 0.01) in graphomotor ability between the different language groups. A post-hoc Bonferroni analysis showed that Hebrew speakers (*n* = 62; z score: M = −0.37, SD = 0.14) achieved significantly lower scores than Arabic speakers (*n* = 31) on the GIFT (z score: M = 0.45, SD = 0.15; *p* < 0.01). There was also a significant difference (*p* > 0.05) between Hebrew speakers and bilingual children (*n* = 36) (z score: M = 0.20, SD = 0.1), with the Hebrew speakers again scoring significantly lower. Participants who were bilingual mostly spoke Arabic and Hebrew, or Hebrew and English.

### 3.2. Microsystem

The study included two components of the microsystem that reflect the home environment: frequency and independence in literacy activities at home, and housing density as an indicator of the physical environment at home. Significant positive Pearson’s correlations were found between GIFT score totals and HLEQ scores both in frequency and independence. There was no correlation between housing density and GIFT total scores (see Table 4).

### 3.3. Exosystem

At the exosystem level, educational approaches were included. Based on Welch’s *t*-test for unequal variance, a significant difference was found in the GIFT total score, where children who attended public education received higher scores than those attending Waldorf preschools (see Table 3).

### 3.4. Macrosystem

Macrosystem-level factors include immigration status, which reflected the cultural background of the parents; and maternal education, which reflected their SES. Based on Welch’s *t*-test for unequal variance, we found no significant difference between the GIFT total scores of children of parents who were foreign-born (at least one of the parents migrated to Israel) and those of Israeli-born parents (see Table 3). Additionally, no significant Pearson’s correlation was found between the GIFT total scores and the mothers’ years of education (see Table 4).

### 3.5. Prediction of Graphomotor Skills

We conducted hierarchical regression analyses in order to examine factors contributing to the predictive ability of GIFT total scores. Only variables showing significant differences or correlation coefficients were included. The entire model was significant (F_(6,126)_ = 15.92, *p* < 0.001) explaining 40.4% of the variance (R^2^ = 0.431, adjusted R square = 0.404). The model indicated that after controlling for personal factors of gender, age, and language, the frequency scale of the HLEQ accounted for 7.9% (*p* = 0.024) of the variance, and the anthroposophical (Waldorf) educational approach accounted for an additional 17.2% (*p* < 0.001). The results are summarized in Table 5.

## 4. Discussion

**Figure 1:**
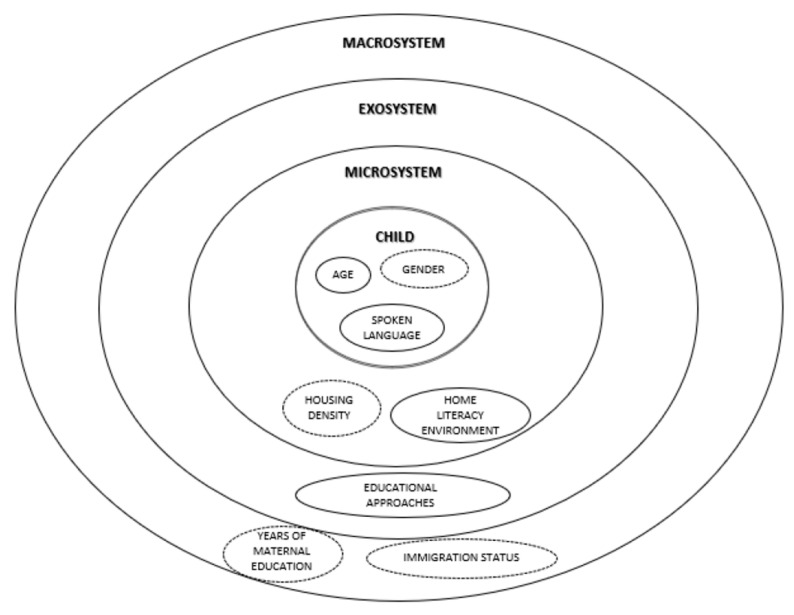
Bronfenbrenner’s bioecological model of human development (Notes. Variables located in the model that are significant to graphomotor skills are enclosed in solid lines; nonsignificant variables are in broken lines).

This study investigated the influence of environment and personal factors on a child’s graphomotor skills during the preschool years. The results of our study, summarized in Figure 1, generally agreed with the bioecological design model [23] indicating that the relationship between the environment and a child’s graphomotor skills is strongest in the proximal circle, specifically at home and in school; whereas it was not evident in the distal system [53]. Bronfenbrenner’s model is deemed most useful since it enables examination of the interaction between the growing child and the environment at all levels [54]. 

Regarding personal factors, there was a positive correlation with age only for the two youngest age groups, which supports the hypothesis that although children’s repertoire of motor skills leaps forward during their preschool years, by age five, a child has well-developed eye-hand coordination [10]. The current study did not reveal gender-related differences in girls’ and boys’ skills, in contrast to previous graphomotor studies [48,55,56]. Our contradictory findings suggest that gender differences in motor performance are either too small to be easily detected, or that they are restricted to specific motor skills, some of which favor girls while others favor boys [11].

We found that bilingual children had better graphomotor skills than those who spoke Hebrew only. Our results are in line with a previous study that compared drawings in four- and five-year-old bilingual children (English–Hebrew and Arabic–Hebrew), compared to their monolingual peers (*n* = 80). Those results revealed that bilingual children inserted significantly more features of other representational categories into their drawings, suggesting that bilinguals’ language experiences may advance a type of representational cognitive flexibility [57].

Arabic speakers scored higher than Hebrew speakers. While Arabic and Hebrew are Semitic languages and share several linguistic aspects, they differ greatly in terms of letter forms, spatial organization, and orthographic complexity [58]. The current results may be explained by the complicated characteristics of the Arabic alphabet, in which letters change form based on whether they are in the beginning, middle, or end of a word [59]. Furthermore, various Arabic letters have a similar or even identical shape and can only be distinguished by the number, location, and size of dots [60]. In light of these findings, learning the Arabic alphabet may facilitate better graphomotor performance [61].

The most noteworthy finding, that can be incorporated into early intervention programs, was the positive correlation between a child’s graphomotor skills and family literacy experiences. Specifically, the frequency scale was a significant predictor of graphomotor skills. Previous studies have also found a positive correlation between literacy and graphomotor ability [1,62,63]. For example, a study analyzed 80 first-grade students and found that copying geometric figures played a significant role in children’s awareness of print and reading abilities [64]. Generally speaking, preschool is a crucial period of time for children to develop motor and literacy skills [65]. Adults can support the development of children’s early literacy in different ways and during various activities within the immediate environment of the child [66], increasing their readiness for primary education [65].

A significant performance difference was found between children who attended public preschool and those who attended the private (Waldorf) preschool. Moreover, the preschool curriculum was a significant predictor of graphomotor skills. This finding indicates that different educational experiences have an impact on child development. Our results are consistent with a previous study that found Waldorf pupils performed less well than mainstream students in free and realistic drawings of people, houses, or objects. However, children who attended Waldorf schools produced more expressive drawings when using facial expressions [67]. These results can be explained by the Waldorf curriculum, which avoids direct guidance in graphomotor skills like drawing, until the age of 12 [67]. Accordingly, previous research has recommended that teachers should be more intentional and specific in fine motor interventions with preschoolers, in order to prevent negative outcomes and fine motor difficulties [68]. It is important for early childhood educators to provide students with materials and activities to facilitate the use of their fingers and hand muscles [21]. Additionally, the GIFT battery may better match the curriculum of public kindergartens as published by the Israeli Ministry of Education [69].

There was no association between the macrosystem environment elements (SES and immigration status) and graphomotor skills. Our results are contrary to studies that have found that children from a high SES perform significantly better in both fine- and gross-motor skills than children from a middle or low SES [55,70,71,72]. Our results might be explained by the relatively high SES of our sample, with more than 70% of mothers receiving more than 12 years of education, in contrast to an opposite ratio presented in other studies [73]. It is therefore hard to tell whether the null effect of SES was due to sampling issues or a genuine lack of influence on graphomotor development. The immigration status results are in line with those of a previous study, which found no significant differences in visual-motor development between children born to Italian parents in Italy and those born to foreign parents in Italy, suggesting that children raised in the same cultural context have similar training paths [74]. Another study showed that parental immigration status had a negative impact on child development only when the immigrant parents came from low-income families [39], which was not the case in our sample.

### Limitations and Future Directions

As Bronfenbrenner and Evans (2000) previously noted, the bioecological model is not a theory about how human beings develop, but rather, it aims to improve our understanding of the conditions and processes that influence human development [24]. Moreover, understanding the relationship between environmental factors and preschool motor skills is useful for both educators and clinicians in order to implement context-sensitive early intervention action [75]. In that regard, we believe that our current findings have furthered our knowledge about the contextual conditions as well as personal characteristics that influence children’s graphomotor development. Nevertheless, some limitations should be taken into account when interpreting these findings. First, we chose to focus on a few factors that represent each environment according to Bronfenbrenner‘s ecological model. The classification of the factors within each system has been controversial due to different interpretations of the model over the years [76]. In addition, in some versions of bio-ecological models, a mesosystem, which includes interactions between his or her microsystems, is also included [77]. Thus, bearing in mind that all the varied aspects of bioecological model theory cannot be examined in a single investigation [25], it was, however, not included in this study. To better understand the potential implications of the bioecological model for graphomotor as well as other motor skills, future studies should examine additional personal and environmental factors. Furthermore, although our convenience sample benefited from the ethnic and geographic diversity of Israel, the SES of the participants was relatively high, with only 17.7% of the mothers having 12 years or less of education. A more diverse range of SES backgrounds is therefore needed for future studies. Lastly, we limited our motor competence investigation to graphomotor skills only, by using GIFT total scores. We also evaluated the environment based on demographic and literacy data from parents. Future studies should use objective and subjective tools to assess additional motor skills and environmental characteristics. 

## 5. Conclusions

Congruent with Bronfenbrenner’s (1979) theory, our findings shed light on the relationship between specific personal and environmental factors and graphomotor skills in typically developing young children by demonstrating the relationships of different bioecological circles centered around the child. The development of graphomotor skills was predicted by age and spoken language, as well as by home literacy activities and educational approaches. However, there were no significant associations with distal environments, such as SES or parental immigration history. This study aligns with contemporary views of early childhood motor development, which emphasize the importance of the environment in determining growth and development [13,20].

## Figures and Tables

**Table 1:** Socio-demographic characteristics of the sample (*n* = 136).

Variable	*n* (%)	*M* (*SD*)
Child’s age		5.15 (1.06)
Gender		
Boys	66 (48)	
Girls	70 (52)	
Educational setting		
Daycare	11 (8.27)	
Pre preschool	53 (39.8)	
Preschool	35 (26.3)	
School	34 (25.6)	
Educational system		
Public	92 (71)	
Waldorf	37 (29)	
Years of maternal education		
Above 12 years	105 (80.7)	
12 years and below	25 (19.2)	
Housing density ^a^		1.17 (0.36)
Spoken language(s) at home (*n* = 134)		
Hebrew	62 (49.6)	
Arabic	31 (22.8)	
Bilingual	36 (26.8)	
Others (Russian and English)	5 (3.7)	
Residential type		
Urban	75(55.9)	
Rural	59 (44.02)	

Note. ^a^ Housing density = persons per room.

**Table 2:** Descriptive information: GIFT and HLEQ.

Variable	*n*	*M* (*SD*)	Range	Correlation with Age
GIFT total score				
Age (years: months)				
3–4	16	14.19 (3.87)	0–20	0.7 **
4–5	42	25.26 (7.84)	0–35	0.55 ***
5–6	47	38.87 (8.88)	0–50	0.1
6–7	31	38.61 (8.73)	0–50	0.3
HLEQ			Scales	
Frequency scale	135	3.26 (1.04)	1–6	
Independence scale		3.09 (0.98)	1–5	

Notes. GIFT: Gilboa Functional Test; HLEQ: Home Literacy Experiences Questionnaire. ** *p* < 0.01, *** *p* < 0.001.

**Table 3:** Personal and environmental factors: differences in the performance of GIFT.

Variable	*n*	GIFT Total Score *M* (*SD*)	*t*	Cohen’s d
Personal			1.74	0.29
Gender				
Boys	66	−0.15 (1.03)		
Girls	70	0.14 (0.92)		
EXOSYSTEM			8.45 **	1.54
Educational approach				
Waldorf	37	−0.95 (0.92)		
Public	99	0.35 (0.75)		
MACROSYSTEM			0.71	0.55
Parental immigration status				
Foreign-born	50	0.08 (0.87)		
Born in Israel	84	−0.45 (1.04)		

Notes. GIFT: Gilboa Functional Test; total scores are presented as z scores. ** Difference is significant at the 0.01 level (two-tailed).

**Table 4:** Pearson correlation coefficients between the GIFT total scores and the environmental factors.

Variable	*n*	r
HLEQ Frequency	135	0.37 **
HLEQ Independence	134	0.34 **
Housing Density	133	−0.08
Mothers’ years of education	127	−0.09

Notes. GIFT: Gilboa Functional Test; HLEQ: Home Literacy Experiences Questionnaire. ** *p* < 0.01, two-tailed.

**Table 5:** Regression analysis for GIFT total score with associated factors.

Variable	Cumulative		Simultaneous	
	*R*² Change	*F*-Change	*β*	*p*
Block 1: personal factors	0.180	9.417 ***		
Age			0.185	0.026
Gender			−0.146	0.070
Hebrew language			−0.397	0.000
Block 2: microsystem	0.079	6.809 **		
Literacy at home: HLEQ				
Frequency scale			0.235	0.024
Independence scale			0.100	0.331
Block 3: exosystem	0.172	38.129 ***		
Educational approach:				
Anthroposophical approach			−0.544	0.000

Note. HLEQ: Home Literacy Experiences Questionnaire. ** *p* < 0.01, *** *p* < 0.001.

## Data Availability

The data that support the findings of this study are available on request from the corresponding author.
